# The Quiet Burden of Iron: A Rare Case of Hereditary Hemochromatosis in Pakistan

**DOI:** 10.7759/cureus.88355

**Published:** 2025-07-20

**Authors:** Maha Batool, Noor Ehsan, Muhammad Imran, Wafa Qaisar, Mahmood N Malik

**Affiliations:** 1 Internal Medicine, Gulab Devi Teaching Hospital, Lahore, PAK; 2 Internal Medicine, Gulab Devi Teaching Hospital/Al-Aleem Medical College, Lahore, PAK

**Keywords:** bronze diabetes, heart failure, hereditary hemochromatosis, hypogonadotropic hypogonadism, iron overload, liver cirrhosis

## Abstract

Hereditary hemochromatosis is characterized by excessive iron absorption through the gut and its deposition in the body. In many instances, symptoms do not arise until significant organ damage has occurred. Diagnosing a case of hereditary hemochromatosis is difficult even after the appearance of symptoms due to the diverse clinical presentations. Genetic testing should be done, whenever possible, to determine the underlying genetic abnormality. We present the case of a 42-year-old man who came to us with complaints of generalized body swelling and shortness of breath on exertion. He was found to have liver cirrhosis, dilated cardiomyopathy, and heart failure with reduced ejection fraction, bronze diabetes, and hypogonadotropic hypogonadism. He was diagnosed with hereditary hemochromatosis based on the clinical findings, markedly elevated serum ferritin levels (>2000 ng/dl), transferrin saturation (98%), and imaging, which revealed pathology involving the liver, pancreas, spleen, and the pituitary gland. Genetic testing could not be done due to the scarcity of resources. The patient underwent phlebotomy initially but was later put on an oral iron chelator, deferasirox, due to a drop in hemoglobin levels. This publication highlights the diagnostic challenge posed by hereditary hemochromatosis, particularly in regions with low disease prevalence, and the need for early detection to prevent advanced organ dysfunction.

## Introduction

Hereditary hemochromatosis is a common inherited disorder among individuals of European descent, affecting 1 in 300 to 500 individuals [1]. However, it is less common in the Asia-Pacific population, probably due to under-recognition and under-reporting [2]. Hereditary hemochromatosis not only affects the liver but also has the potential to cause heart failure, hypothyroidism, hypogonadism, bronze discoloration of the skin, diabetes, arthritis, and arthropathy [3]. Mutations in the hereditary hemochromatosis protein (HFE) gene are most commonly associated with hemochromatosis in the Caucasian population, and these mutations are inherited in an autosomal recessive pattern [4]. In South Asian populations, HFE gene mutations are reported, with p.H63D being more prevalent than p.C282Y, and H63D mutation frequencies ranging from 8% to 92% [5]. Other genetic mutations exist, namely HFE2, HAMP, TFR2, and SCL40A1, the latter being the only mutation inherited in an autosomal dominant fashion [1]. A mutation in the HFE gene causes dysregulated hepcidin function, resulting in decreased hepcidin levels, which leads to increased iron absorption and, ultimately, iron overload [6]. Diagnosing a case of hemochromatosis may prove to be challenging due to the diverse range of manifestations, as multiple organ systems can be affected [7]. We present this case of primary hemochromatosis to highlight the diagnostic challenge clinicians face. We also want to stress the significance of a timely diagnosis, as early intervention could mitigate the risk of complications such as liver cirrhosis and heart failure [8,9].

## Case presentation

A 42-year-old male presented to the Medical Outpatient Department of Gulab Devi Teaching Hospital, Pakistan, in April of 2025, with complaints of generalized body swelling and shortness of breath on exertion for two weeks. The patient initially noticed facial puffiness in the morning, which was followed by limb swelling. This was accompanied by shortness of breath, which initially occurred on climbing stairs but later worsened and led to a limitation of his day-to-day activities. There was no history of chest pain, orthopnoea, paroxysmal nocturnal dyspnoea, hematemesis, melena, or any urinary abnormality. The patient also complained of easy bruising, a change in skin color, loss of body hair, and decreased libido that he had noticed over the past year. The patient was diagnosed with diabetes mellitus a year ago, based on routine blood sugar testing. He was on premixed human insulin 70/30 (70% NPH and 30% regular insulin) and had poor control of his blood sugar levels. He had not undergone any surgeries or blood transfusions. Family history was significant for diabetes mellitus in both parents. He had three siblings; all were alive and healthy.

**Figure 1:**
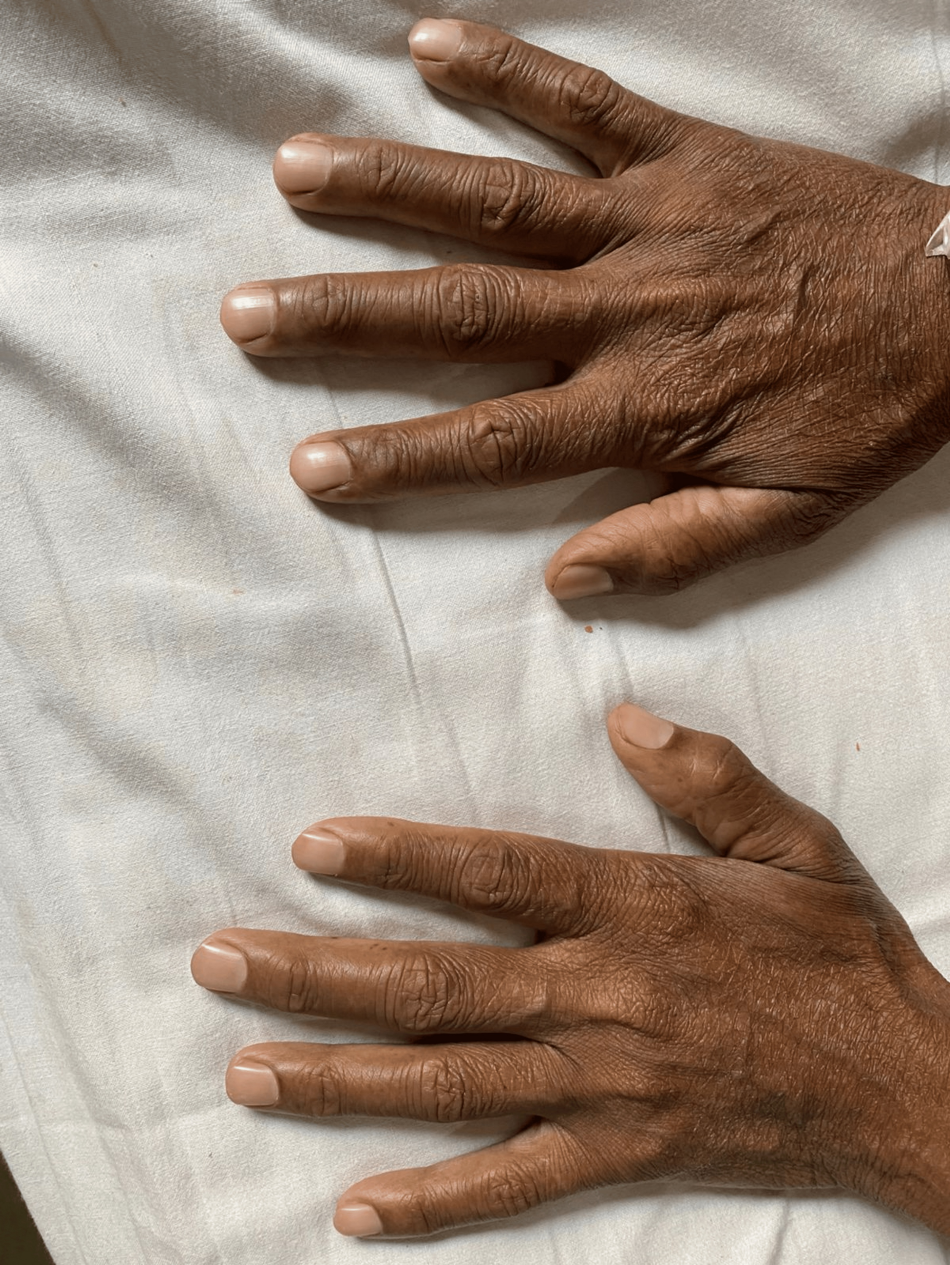
Dorsum of hands showing bronze discoloration of skin and shiny nails.

**Figure 2:**
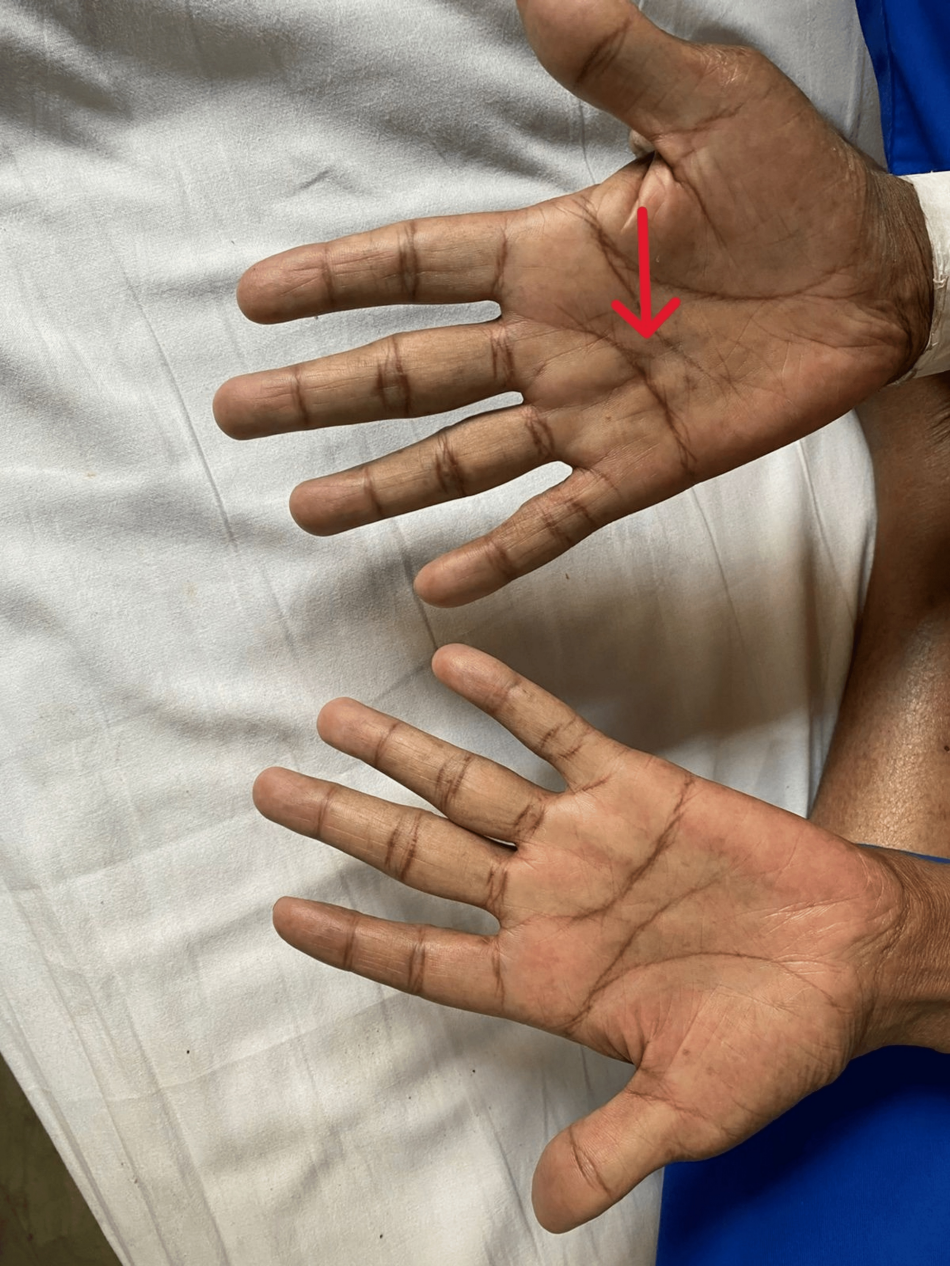
Palmar surface of hands showing palmar erythema bilaterally; the arrow shows Dupuytren’s contracture on the right palm.

**Figure 3:**
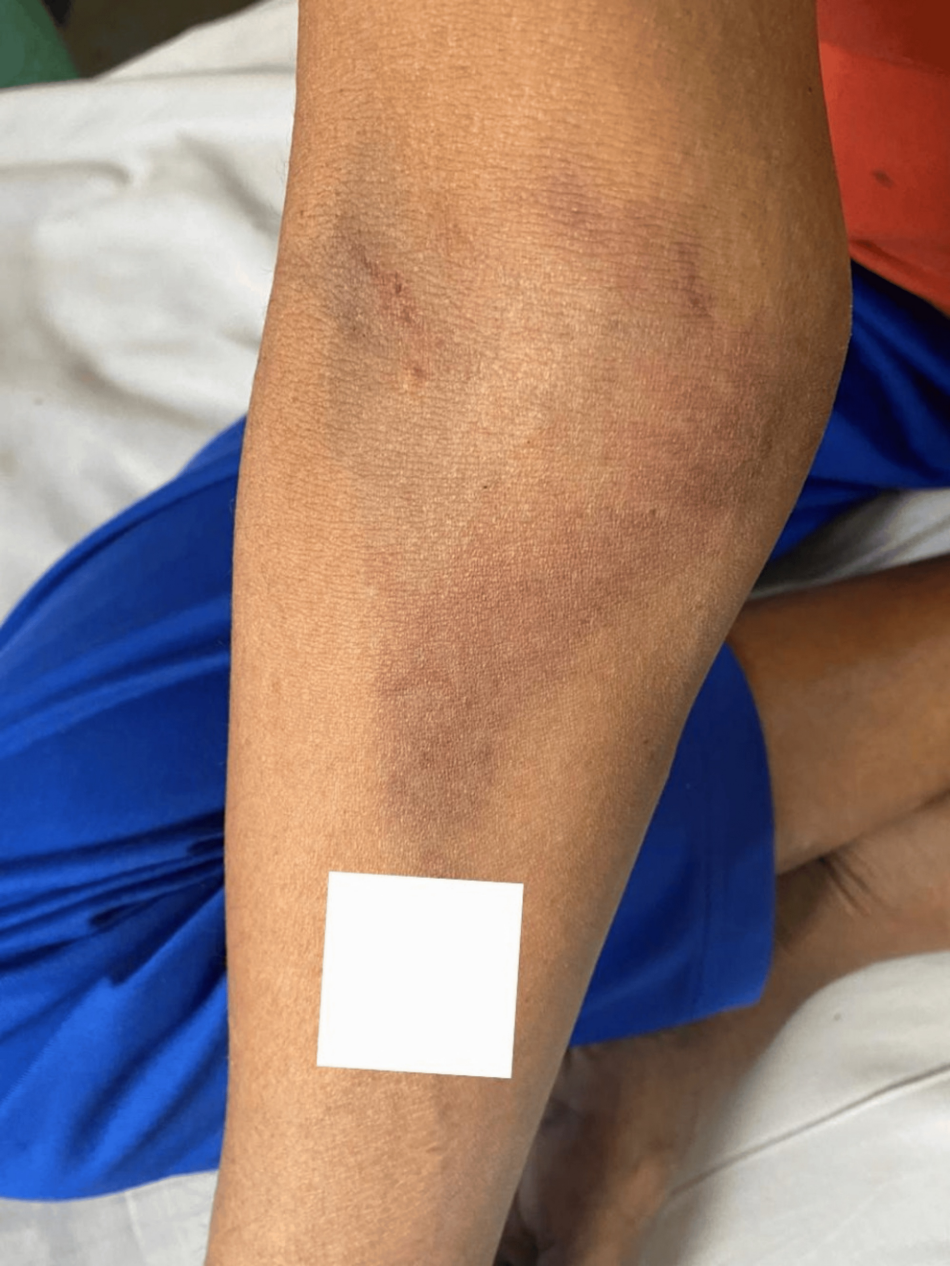
Bruise on the right forearm and elbow. Tattoo mark digitally concealed to protect patient identity.

On examination, he had hyperpigmented skin, shiny nails, palmar erythema, Dupuytren’s contracture on the right palm, and several bruises on his upper limbs (Figures 1-3). His jugular venous pressure was raised. Chest examination revealed decreased breath sounds at both lung bases. A holosystolic murmur was auscultated at the mitral area. He had hepatosplenomegaly. The lower edge of the liver was palpable three cm below the right costal margin, and the spleen was palpable six cm below the left costal margin. Shifting dullness was also appreciated in the abdomen. He had pitting edema extending to his shins bilaterally.

**Figure 4:**
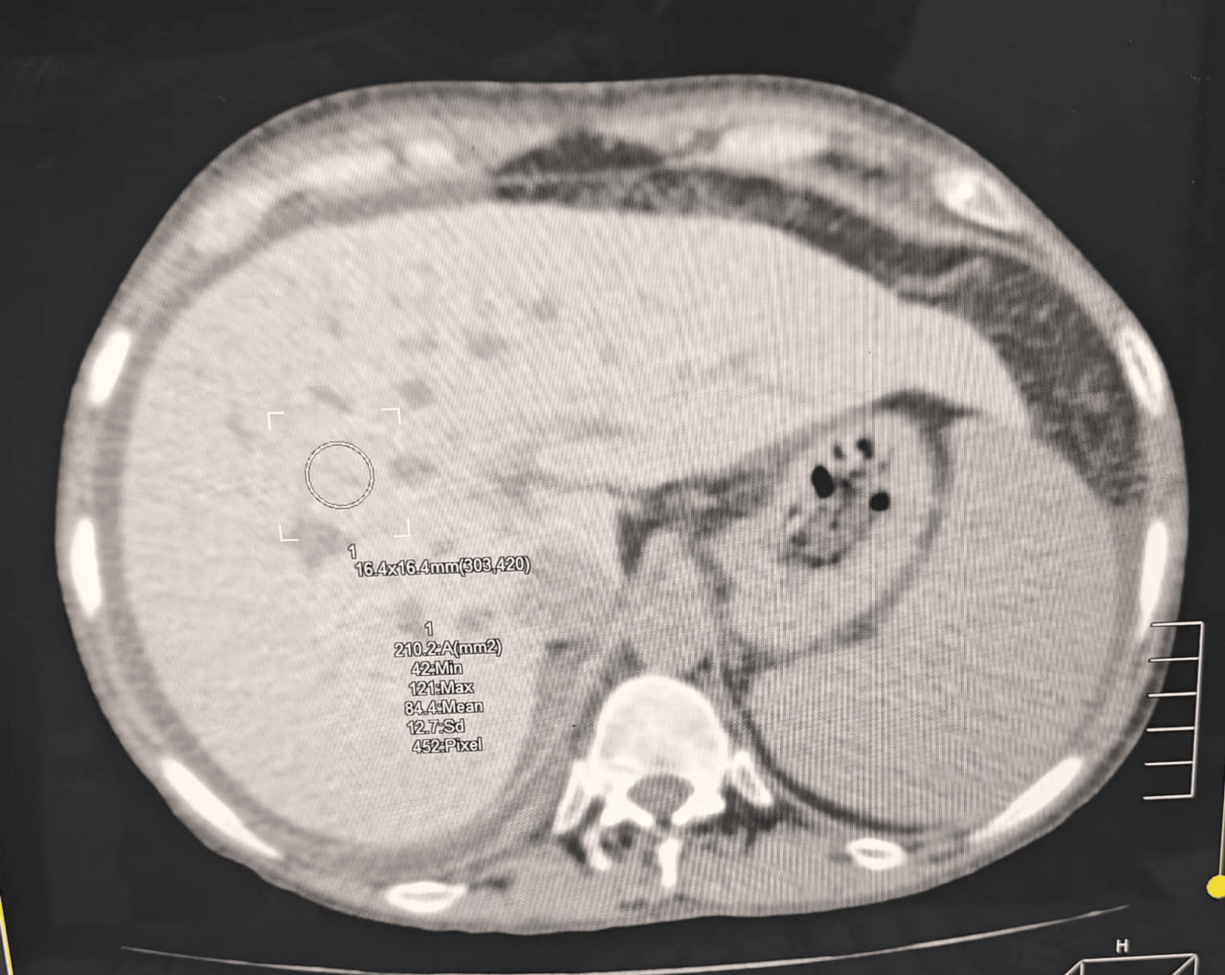
CAT scan of the abdomen and pelvis, the liver shows diffusely increased density, with a mean density of about 85-90 HU. A slight irregularity of the hepatic surface contour is also noted. The portal vein appears normal. CAT scan: Computed axial tomographic scan.

**Figure 5:**
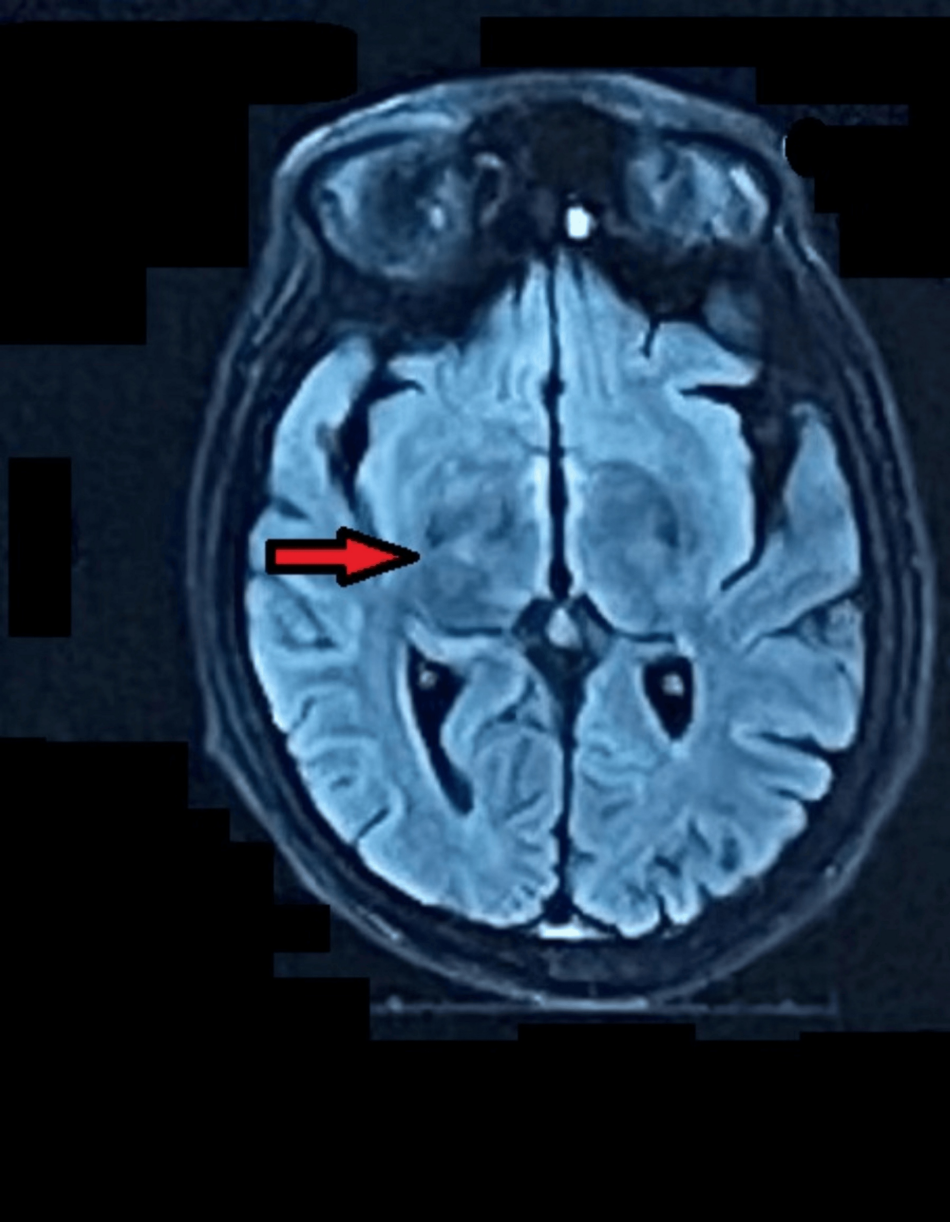
T1-weighted image of an MRI brain with contrast showing iron deposits in the basal ganglia, with patient identifying information digitally concealed in the image. Magnetic resonance imaging (MRI) of the brain with contrast showing hyperintense signals in the region of the basal ganglia, secondary to iron deposits, indicated by the red arrow. The image shows normal grey and white matter distribution. The ventricular system and extraventricular, CSF spaces appear to be normal.

His blood work revealed low hemoglobin and platelet levels. His liver function tests were deranged, and prothrombin time/international normalized ratio (PT/INR) was increased (Table 1). Screening for chronic hepatitis B and C virus infection was negative. The peripheral smear showed normochromic, normocytic red cells. The retic count was 2.1%. His iron studies were done (Table 2). His serum ferritin was more than 2000 ng/dl, and transferrin saturation was 98%. His serum testosterone, follicle-stimulating hormone (FSH), and luteinizing hormone (LH) levels were low (Table 3). His echocardiogram showed evidence of dilated cardiomyopathy, mild mitral and tricuspid valve regurgitation, and a left ventricular ejection fraction of 40%. An abdominal ultrasound was done, which showed a coarse liver echotexture, a dilated portal vein, an enlarged spleen, and mild abdominopelvic ascites. Doppler ultrasound of the testes revealed bilateral testicular atrophy. A computed tomography (CT) scan of the abdomen and pelvis was done, which showed the liver to have irregular surface contours and diffusely increased density of around 85-90 HU (Figure 4). The mean density of the spleen and pancreas was also increased. Brain magnetic resonance imaging (MRI) with contrast showed hypointense signals in the pituitary gland, basal ganglia, and the dentate nuclei on the T2 images, and hyperintense signals in the basal ganglia on the T1-weighted sequences, depicting iron deposits in the aforementioned structures (Figure 5).

**Table 1:** Complete blood count and liver function tests. CBC: Complete blood count, SGPT (ALT): Serum glutamic pyruvic transaminase (also known as alanine aminotransferase), SGOT (AST): Serum glutamic oxaloacetic transaminase (also known as aspartate aminotransferase), ALK: Alkaline phosphatase

Investigation		Patient Value	Normal Range
CBC:			
Hemoglobin		12.6g/dl	13-18 g/dl
Platelet Count		54,000	150,000-450,000
White Blood Cell Count		11.8*10^3/uL	(4-11) *10^3/uL
Liver Function Test:			
Bilirubin		1.0mg/dl	0.02-1.01mg/dl
SGPT (ALT)		64U/L	5-42 U/L
SGOT (AST)		109U/L	5-45 U/L
ALK. Phosphate		340U/L	80-306 U/L

**Table 2:** Serum iron, ferritin, and folate levels. TIBC: Total iron binding capacity, T SAT%: Transferrin saturation percentage.

Iron Studies:		
Investigation	Patient Value	Normal Value
Folate	2.6ng/dl	3.1-20.5 ng/dl
Iron Level	147	59-158 ug/dl
Ferritin	>2000 ng/mL	24-336 ng/mL
TIBC	150ug/dl	250-400 ug/dl
TSAT%	98%	20-50%

**Table 3:** Hormone profile showing serum testosterone, FSH, and LH levels. FSH: Follicle-stimulating hormone, LH: Luteinizing hormone.

Hormonal Profile:		
Investigation	Patient Value	Normal Value
Testosterone	<2.50 ng/dl	249-836 ng/dl
LH	<0.12 IU/L	0.57-12.07 IU/L
FSH	<0.11 IU/L	0.95-11.95 IU/L

Based on the above investigations, the patient was diagnosed with hereditary hemochromatosis. During the patient's hospital stay, twice-weekly phlebotomy sessions were done. 450 ml of blood was removed and donated each time. Unfortunately, phlebotomy had to be discontinued as his hemoglobin levels dropped to 9 gm/dl. Weight-based oral iron chelation therapy started with deferasirox (20 mg/kg body weight, once daily). The patient was also put on injectable testosterone, 125 mg once a month, for six months, to be followed by 250 mg each month. Oral losartan, 25 mg once daily, was also initiated. The patient is on follow-up in the Outpatient Department, with serial CBCs, serum ferritin levels, and transferrin saturation. An upper GI endoscopy is also planned to be done in the next six months to look for the presence of varices. We also plan to screen him for hepatocellular carcinoma by liver ultrasound and alpha-fetoprotein levels every six months. He is also following up with the Cardiology Outpatient Department for the management of his heart failure, with a plan to get a cardiac MRI and to repeat echocardiography every six months.

## Discussion

Hereditary hemochromatosis involves increased iron absorption from the gastrointestinal tract and its accumulation in different organs of the body, like the liver, heart, pancreas, gonads, and skin. The deposited iron leads to oxidative stress, cell damage, and organ dysfunction [3]. Thus, primary hemochromatosis can manifest with a multitude of organ dysfunctions, including chronic liver disease, heart failure, insulin-dependent diabetes mellitus, discoloration of skin, hypogonadism, and arthritis [10]. Clinical manifestations of hereditary hemochromatosis typically occur after the age of 40 in males and 50 in females, the delayed presentation in women being secondary to menstrual blood loss [11]. Our patient’s age of presentation was typical for that of a male presenting with hereditary hemochromatosis. He had never received any blood transfusions. He possessed all the classic features of hemochromatosis. The patient was first suspected of having hemochromatosis when bronze skin discoloration was noted in the presence of heart failure with reduced ejection fraction, insulin-dependent diabetes mellitus, and chronic liver disease. He also had a history of sexual dysfunction and decreased libido, which was later confirmed to be due to hypogonadotropic hypogonadism.

Hereditary hemochromatosis, type 1, is caused by HFE gene mutations. More than 100 such mutations have been identified. The most common HFE gene mutations are p.Cys282Tyr or C282Y and p.His63Asp or H63D. The mutant HFE genes result in either low levels of hepcidin in the body or the development of hepcidin insensitivity, leading to increased iron absorption. Hereditary hemochromatosis types 2a and 2b are caused by mutations in the HFE2 and hepcidin antimicrobial peptide (HAMP) genes. Type 3 is caused by a mutated transferrin-receptor gene (TFR2). Types 1 to 3 are inherited in an autosomal recessive fashion. Type 4 is the only subtype that is inherited in an autosomal dominant fashion, resulting in ferroportin dysfunction due to the mutated SCL40A1 gene [1]. In our case, a genetic workup was not done. 

The diagnosis of hereditary hemochromatosis was made based on elevated serum ferritin levels, transferrin saturation, and liver imaging. Secondary hemochromatosis was excluded based on the absence of a history of blood transfusions, and the peripheral blood smear showed no abnormal erythrocyte morphology. A diagnosis of hemochromatosis should be strongly suspected if the serum ferritin levels rise above 300 ng/dl in a male or above 200 ng/dl in a female. Serum transferrin saturation above 40% when coupled with a markedly raised serum ferritin, is also strongly indicative of hemochromatosis [12]. Our patient’s serum ferritin was greater than 2000 ng/dl, and transferrin saturation was 98%. A computed axial tomography (CAT) scan of the abdomen showed diffusely increased liver density along with evidence of cirrhosis. It also showed evidence of iron deposition in the spleen and pancreas, demonstrated by the increased mean density of the two organs. Multi-organ involvement was also evident by signs of heart failure on echocardiography, which showed a left ventricular ejection fraction of 40%, along with dilated heart chambers and mitral and tricuspid valve regurgitation. Pancreatic involvement was evident from the presence of insulin-dependent diabetes mellitus and the absence of anti-GAD antibodies. Iron deposition in the pituitary gland and the development of hypogonadotropic hypogonadism were confirmed by the low levels of testosterone, follicle-stimulating hormone, and luteinizing hormone; hypointense signals in the pituitary gland on T2-weighted brain MRI; and bilateral testicular atrophy on Doppler ultrasound.

The treatment of choice for hereditary hemochromatosis is phlebotomy [13]. Phlebotomy reduces the iron load in the body, thereby reducing the risk of liver cirrhosis, the development of cardiomyopathy, and diabetes [14]. Oral iron chelators like deferasirox, if initiated early in the disease, could also significantly reduce disease morbidity and improve survival by preventing and reversing cardiomyopathy [15]. 

A limitation of this case report is the lack of genetic testing to confirm the mutation leading to hereditary hemochromatosis. The diagnosis was based on clinical presentation, biochemical evidence of iron overload, and imaging suggestive of multiorgan involvement. We were unable to perform gene testing due to financial limitations.

## Conclusions

This case highlights the importance of considering hereditary hemochromatosis in patients with unexplained liver dysfunction, heart failure, and seemingly unrelated clinical manifestations. Clinicians should maintain a high degree of suspicion, particularly in regions where the disease is underrecognized, as early diagnosis and treatment can prevent complications like liver cirrhosis, heart failure, and diabetes. Biochemical markers like serum iron levels, serum ferritin, and transferrin saturation, and imaging could be used to establish the diagnosis when genetic testing is unavailable or not possible. First-degree relatives of the affected individuals should also undergo screening for the detection of the disease.
